# Buildings Contemplated and in Progress

**Published:** 1914-06-20

**Authors:** 


### Buildings Contemplated and in Progress.

Ashington.—The foundation-stone of a new hospital to be built by the Ashington and District Nursing Association at a cost of about £8,000 has been formally laid. The plans are by Mr. G. Beattie, surveyor to the Urban District Council.

Bournemouth.—The Corporation have approved plans for additions to the Boscombe Hospital.

Bristol.—The Health Committee of the Corporation of Bristol invite tenders for the erection of a consumptive sanatorium at Ham Green. Bills of quantities may be obtained of the city engineer, Lessel S. McKenzie, A.M.Inst., 63 Queen's Square, Bristol.

Chester.—Tenders are invited for the erection of a tuberculosis ward adjoining the Isolation Hospital, Sealand, Chester. Quantities to be obtained of the city surveyor, Town Hall, Chester.

Durham.—Plans have been approved for stores and bathrooms at the County Hospital.

East Ham.—The Town Council have passed plans and estimates, amounting to £11,392, for isolation hospital extensions.

Hanwell.—The Urban District Council Public Health Committee are considering plans prepared by the surveyor for an isolation hospital on a site at the sewage farm.

Hemel Hempstead.—The Hemel Hempstead Joint Hospital Board invite tenders for the erection of an isolation hospital at Bennet's End, Hemel Hempstead. The architects are Messrs. Saxon, Snell and Spoor, of 26 Great James Street, Bedford Row, W.C., from whom forms of tender and quantities may be obtained.

Hitchin.—The Rural District Council invite tenders for the erection of an isolation hospital, with the necessary administration block, laundry, etc. Mr. H. Percy Adams, 28 Woburn Place, Russell Square, W.C., is the architect. Quantities and forms of tender may be obtained of the clerk to the Council, Mr. A. E. Passing Lane, 5 Bancroft, Hitchin, Herts.

Norton.—The Stockton Guardians have decided to apply to the Local Government Board for sanction to purchase $5\frac{1}{2}$ acres of land in Junction Road for a new infirmary.

Samford.—The Samford Rural District Council invite tenders for additions and alterations at the Infectious Diseases Hospital at Tattingstone. Particulars may be obtained from Mr. Arnold J. Haward, Clerk, 34 Princes Street, Ipswich.

Ufford.—The East Suffolk County Council have decided to apply for compulsory powers to purchase the Stone Farm land at Ufford for the extension of the County Mental Hospital.

